# Design, Synthesis and Characterization of a Novel Type of Thermo-Responsible Phospholipid Microcapsule–Alginate Composite Hydrogel for Drug Delivery

**DOI:** 10.3390/molecules25030694

**Published:** 2020-02-06

**Authors:** Liang Ding, Xinxia Cui, Rui Jiang, Keya Zhou, Yalei Wen, Chenfeng Wang, Zhilian Yue, Shigang Shen, Xuefeng Pan

**Affiliations:** 1 Medical College, Hebei University, Baoding 071000, China; dingliangzi88@126.com (L.D.); ming3967@sina.com (R.J.); wenyaleiyaoli@163.com (Y.W.); 2 College of Chemistry and Environmental Science, Hebei University, Baoding 071002, China; cxx18832860627@163.com (X.C.); zhoukeya@yeah.net (K.Z.); chenfeng_wang@foxmail.com (C.W.); 3 Intelligent Polymer Research Institute, AIIM Facility, University of Wollongong, Wollongong, NSW 2522, Australia; zyue@uow.edu.au; 4 School of Life Science, Beijing Institute of Technology, Beijing 100081, China

**Keywords:** thermo-responsive liposome, phospholipid microcapsules, sodium alginate, hydrogel, protamine–siRNA complex, drug delivery

## Abstract

Liposomes are extensively used in drug delivery, while alginates are widely used in tissue engineering. However, liposomes are usually thermally unstable and drug-leaking when in liquids, while the drug carriers made of alginates show low loading capacities when used for drug delivery. Herein, we developed a type of thermo-responsible liposome–alginate composite hydrogel (TSPMAH) by grafting thermo-responsive liposomes onto alginates by using Ca2+ mediated bonding between the phosphatidic serine (PS) in the liposome membrane and the alginate. The temperature-sensitivity of the liposomes was actualized by using phospholipids comprising dipalmitoylphosphatidylcholine (DPPC) and PS and the liposomes were prepared by a thin-film dispersion method. The TSPMAH was then successfully prepared by bridge-linking the microcapsules onto the alginate hydrogel via PS-Ca^2+^-Carboxyl-alginate interaction. Characterizations of the TSPMAH were carried out using scanning electron microscopy, transform infrared spectroscopy, and laser scanning confocal microscopy, respectively. Their rheological property was also characterized by using a rheometer. Cytotoxicity evaluations of the TSPMAH showed that the composite hydrogel was biocompatible, safe, and non-toxic. Further, loading and thermos-inducible release of model drugs encapsulated by the TSPMAH as a drug carrier system was also studied by making protamine–siRNA complex-carrying TSPMAH drug carriers. Our results indicated that the TSPMAH described herein has great potentials to be further developed into an intelligent drug delivery system.

## 1. Introduction

Liposomes are hollow structured vesicles formed by phospholipid molecules [1], which are often used as drug carriers. Liposomes normally have low toxicity and high biocompatibility and can be used for sustained release of drugs [2,3]. A thermosensitive liposome (TSL) exhibits a temperature-dependent phase behavior and remains stable at normal body temperature, but disassembles when the environmental temperature is lower than the phase transition temperature (Tm) [4]. The aliphatic hydrocarbon chains of the phospholipid molecules are closely associated with each other to form a bi-layered membrane, which is a stable state with low permeability, while the phospholipid bilayer membrane becomes loosely structured with a compromised membrane permeability when the temperature is higher than the Tm, thereby the embedded drug can be released [5,6,7,8].

To improve the stability of TSL, modifications on the liposome membrane have been reported using metal ions [9], cholesterol [10], polypeptide [11], polymers including polyethylene glycols (PEG) and their derivatives [12,13,14], or surfactants with similar chemical structures to PEG [15]. For example, polyethylene glycol stearyl ether (Brij78) has been incorporated into liposomes via membrane hydration [16]. The Tm of Brij78–liposome is 41 °C, slightly lower than LTSL.

Sodium alginate (SA) is composed of α-l-mannuronic acid (M) and its C5 isomer β-d-guluronic acid (G) linked by (1, 4) glycosidic bonds, forming linear block copolymers composed of different proportions of GM, MM, and GG fragments [17,18]. SA has been widely used in the biomedical field due to its excellent biocompatibility and good bioresorbability [19]. In particular, SA is rich in hydroxyl groups and carboxyl groups and can be easily crosslinked with some divalent cations to form a hydrogel [20] for applications in drug delivery [21,22].

In this study, a type of thermosensitive phospholipid microcapsule was developed using dipalmitoylphosphatidylcholine (DPPC), l-phosphatidylserine (PS), and Brij78 by a film dispersion method. The thermo-responsible phospholipid microcapsules were then grafted onto SA using Ca^2+^ as a crosslinking agent via forming PS-Ca^2+^-COOH between the PS and the alginate, to yield a novel type thermo-responsible phospholipid microcapsule-alginate composite hydrogel (TSPMAH) [23]. Finally, the system was assessed for loading and release capacities of protamine-siRNA. Our results showed the TSPMAH has not only improved the stability of phospholipid microcapsules, the low drug encapsulation by SA, but also showed basically non-toxic and highly cytocompatible to both endothelial cells and myocardial cells, suggesting a potential of being used in the biomedical field.

## 2. Results and Discussion

### 2.1. Preparation of and Characterization of the TSPMAH

#### 2.1.1. Preparation of Thermosensitive Phospholipid Microcapsules

**Figure 1:**
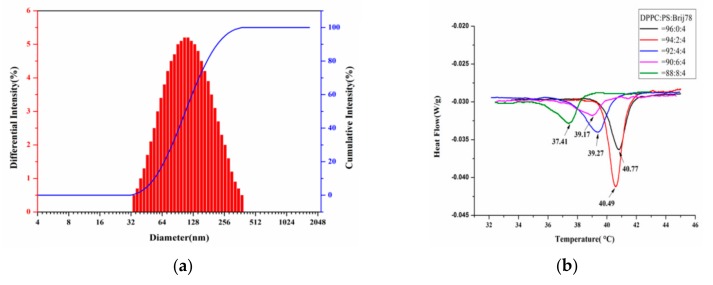

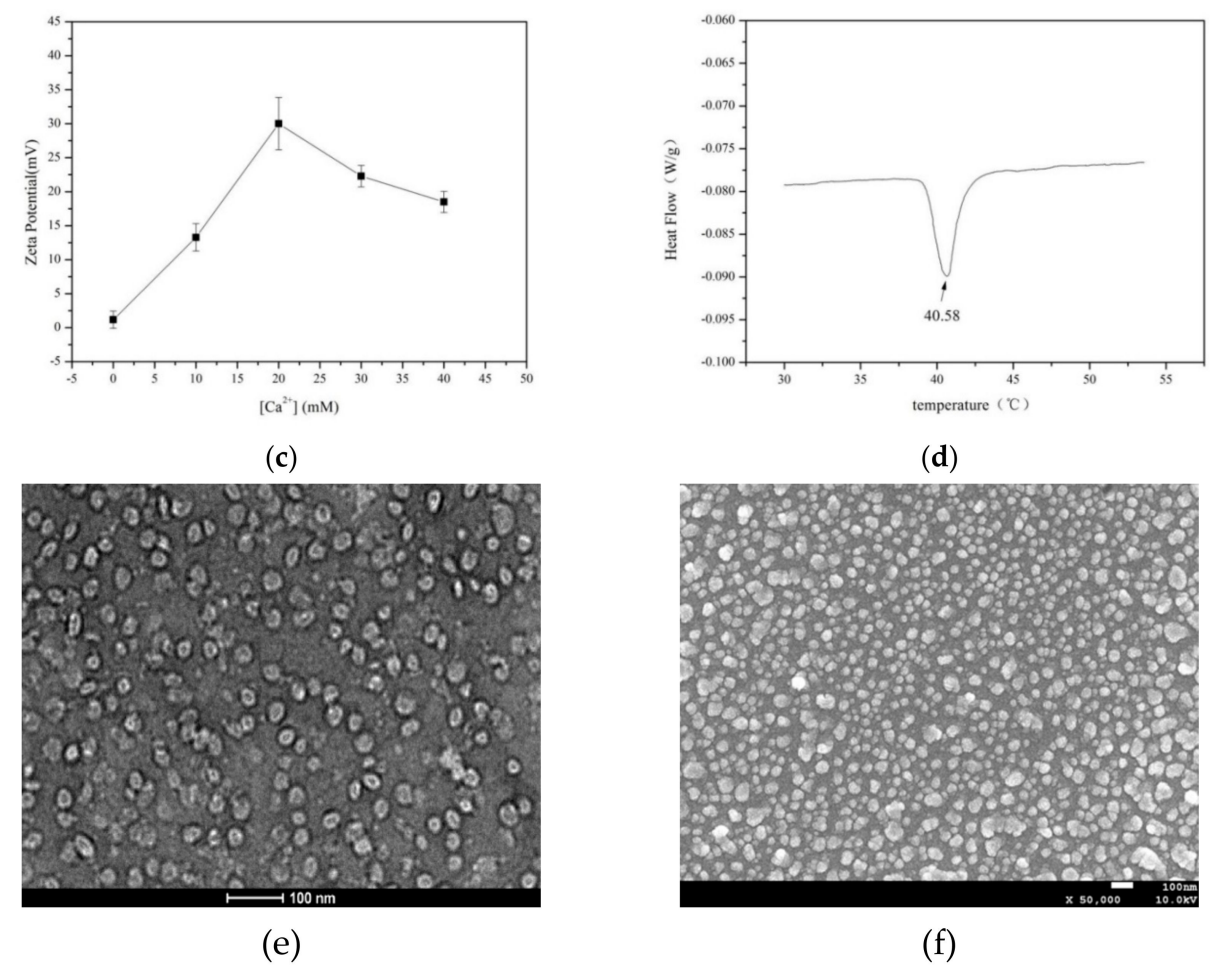
Characterization of the thermosensitive phospholipid microcapsules. Particle size and distribution (**a**), Tm of different dipalmitoylphosphatidylcholine (DPPC):phosphatidic serine (PS):Brij78 molar ratio (**b**), zeta potential at different concentrations of Ca^2+^ (**c**), phase transition temperature (Tm) under the concentration of 20 mM Ca^2+^ (**d**), TEM image (**e**), and SEM image (**f**) of the thermosensitive phospholipid microcapsules.

We prepared a type of temperature-responsible phospholipid microcapsules composed of DPPC, PS, and Brij78, using a widely used membrane dispersion method. The particle sizes of the temperature-responsible phospholipid microcapsules were controlled by adjusting the ultrasonic power of a cell disrupter. The particle sizes of the phospholipid microcapsules were shown to become smaller when the ultrasonic power was increased from 60 w to 180 w. The particle sizes of the phospholipid microcapsules were 220 ± 4.6 nm (Figure 1a). 

Differential scanning calorimetry (DSC) is an important analytical method to investigate the effect of Tm on liposome membrane permeability and liposome structure changes. The Tm of the composite phospholipid was determined as a function of the molar ratio of DPPC:PS:Brij78 (96:0:4, 94:2:4, 92:4:4, 90:6:4, 88:8:4). As shown in Figure 1b, all the samples exhibited temperature-dependent phase behavior. The Tm of the phospholipid microcapsules decreased with increasing the PS content, suggesting that the PS was critical to the phase transition of the phospholipid microcapsules [24,25]. Since PS was thought to be a cell death signal during cell apoptosis in human cells, phagocytic cells would recognize and swallow any phospholipid microcapsules once the PS was exposed outside the phospholipid membrane [26,27]. When the molar ratio of DPPC, PS, and Brij78 was 94:2:4, the optimal Tm of the phospholipid microcapsules was 40.5 °C, slightly higher than the human body. The as-prepared phospholipid microcapsules showed significant temperature response by rapidly releasing the drugs encapsulated in the phospholipid microcapsules. Therefore, the molar ratio of DPPC, PS, and Brij78 was selected to be 94:2:4.

Based upon the electric double layer theory of colloids, when a solution of CaCl_2_ was added, the phospholipid microcapsules formed a colloidal particle with Ca^2+^, and the stability of the suspension was increased due to increased electrostatic repulsion among the colloidal particles [28]. The effects of Ca^2+^ on the sizes and zeta potentials of the temperature-responsive phospholipid microcapsules were investigated (Table 1). As can be seen in Table 1, the particle sizes of the temperature-responsive phospholipid microcapsules were not affected by increasing the Ca^2+^ concentration; however, the zeta potentials, as presented in Figure 1c, were changed by adding Ca^2+^ in the suspension. The maximum zeta potential was seen when the Ca^2+^ concentration was 20 mM (Figure 1c), suggesting the Ca^2+^ complexed phospholipid microcapsules were most stable at this condition.

The Tm of the temperature responding phospholipid microcapsule made up of DPPC: PS: Brij78 = 94:2:4 and 20 mM Ca^2+^ was measured using DSC. The results (Figure 1d) showed that the Tm of the phospholipid microcapsule was at 40.6 °C and not significantly affected by Ca^2+^ complexation. 

Finally, the morphology of the temperature responding phospholipid microcapsules was observed by transmission electron microscopy (TEM) and scanning electron microscopy (SEM). As shown in Figure 1e, the thermosensitive phospholipid microcapsules exhibit a spherical or elliptical membrane structure with good dispersibility and uniformed sizes. The phospholipid microcapsules were independently present, indicating that the microcapsules were in a relatively stable state without significant aggregation (Figure 1f).

#### 2.1.2. SEM and FTIR Characterization of the TSPMAH

**Figure 2:**
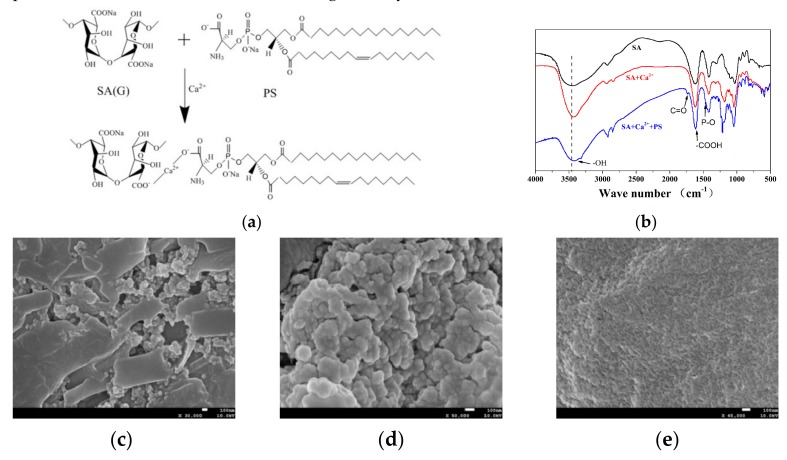
Schematic diagram of the bonding between the PS in thermo-responsive phospholipid microcapsules and the carboxyl groups in the alginate (**a**), FTIR spectra (**b**), SEM images of prepared by addition of microcapsules to sodium alginate (SA) (**c**), SEM images of TSPMAH by addition of SA to microcapsules (**d**), and SEM images of SA crosslinked with Ca^2+^ (**e**).

According to the schematic diagram shown in Figure 2a, a thermo-responsive phospholipid microcapsule–SA composite hydrogel was prepared. The specific functional groups contained in the substance could be known by the FTIR technique to determine whether the complex was successfully synthesized. Figure 2b illustrates the FTIR spectra of SA, SA and Ca^2+^ crosslinking (SA + Ca^2+^), and TSPMAH (SA + Ca^2+^ + PS). There was a distinct broad peak at about 3440 cm^−1^, corresponding to the stretching vibration peak of the hydroxyl groups. The stretching vibration peak of the hydroxyl group had a tendency to move from SA to TSPMAH (SA + Ca^2+^ + PS), indicating that the hydroxyl group on SA was occurring. The reaction might be chelated with Ca^2+^. A small absorption peak appeared at 1466 cm^−1^ in the infrared spectrum of TSPMAH (SA + Ca^2+^ + PS), which was the vibrational absorption peak of P-O-CH_3_ in phospholipid microcapsules [27]. At 1740 cm^−1^ is an asymmetric C=O stretching vibration that was considered to be characteristic of phospholipids [28], and this peak appeared in the TSPMAH (SA + Ca^2+^ + PS). All the characteristics indicated that the thermo-responsive phospholipid microcapsules were successfully grafted onto SA. No new absorption peaks appeared in the spectra of SA and SA-Ca^2+^ crosslinking (SA + Ca^2+^), which was consistent with the hypothesis.

SEM characterization of the TSPMAH showed that the morphology of the TSPMAH formed by different addition methods of SA was slightly different. When the thermo-responsive phospholipid microcapsules were added to SA, as shown in Figure 2c, the microcapsules could not be dispersed well in SA, probably because of the excessive amount of SA around the microcapsules just after the addition, and SA was viscous so that the microcapsules were not easily dispersed uniformly. When SA was added into the phospholipid microcapsules, both Ca^2+^ and phospholipid microcapsules around SA were excessive. As a result, the carboxyl group on the G segment of the SA was crosslinked with Ca^2+^ to form an “egg-box” structure [29,30], and according to Figure 2d, sodium alginate could easily capture phospholipid microcapsules by the bonding mode of SA–Ca^2+^–PS due to its lattice-like spatial structure. The TSPMAH prepared by this method overcame the thermodynamic instability of the phospholipid microcapsules and also solved the problem of low drug loading of SA. By comparing with SA crosslinked with Ca^2+^ (Figure 2e), it could be seen that the SA was dropped into the phospholipid microcapsules to form a composite hydrogel better, which provided a better carrier material for drug delivery.

#### 2.1.3. Rheological Behavior of the TSPMAH 

**Figure 3:**
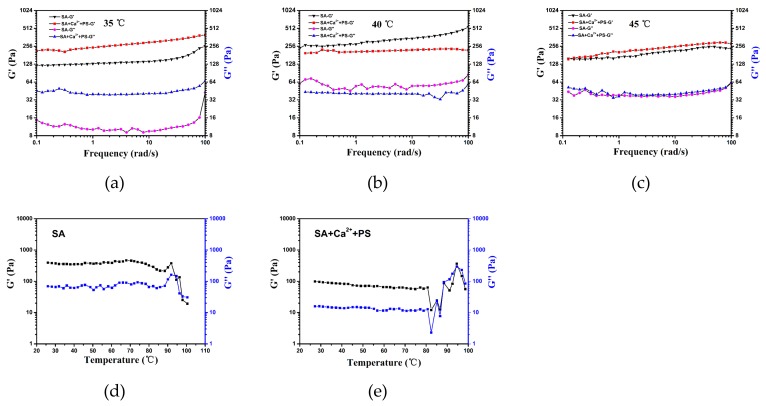
Rheological study of thermo-responsible phospholipid microcapsule-alginate composite hydrogel at 35 °C (**a**), 40 °C (**b**), 45 °C (**c**), and temperature programming between SA (**d**) and SA + Ca^2+^ + PS (**e**).

To understand the effects of the addition of the phospholipid microcapsule on the TSPMAH structure formation and the responses to temperature changes, the rheological behavior of the TSPMAH via temperature changes were analyzed (Figure 3). At 35 °C, 40 °C, and 45 °C, the frequency scanning results showed the G’ of the hydrogel was significantly higher than G’’, suggesting gel-like viscoelastic properties [31]. At 35 °C, the G’ of phospholipid microcapsule composite alginic acid hydrogel was significantly higher than that of calcium alginate gel (Figure 3a), indicating that its crosslinking degree was higher than that of calcium alginate, which might be due to the additional bonding of the carboxyl group of PS in the microcapsules to Ca^2+^. By which, the crosslinking and entanglement of the gels were increased. At 40 °C, the G’ of calcium alginate was slightly higher than the G’ of phospholipid microcapsules combined with alginic acid (Figure 3b), and this is presumably because of reaching the phase transition of phospholipid microcapsules at 40 °C, affecting the crosslinking with SA. At 45 °C, the TSPMAH was basically the same as the G’ of the calcium alginate gel (Figure 3c). At this time, the microcapsules might be completely damaged, and the degree of crosslinking was similar to that of calcium alginate. 

The results of the temperature programming (Figure 3d,e) showed that the TSPMAH and calcium alginate gel maintained a gel state (G′ > G″) in a certain temperature range, and the calcium alginate gel was about 90 °C. A node occurred and a phase transition occurred. The TSPMAH had a junction at about 82 °C, and the junction temperature was gradually increased, which might be because the three-dimensional space of SA was occupied by the heat-sensitive phospholipid microcapsules, resulting in decreased water content when at lower Tm.

#### 2.1.4. Analysis of Swelling Degree of TSPMAH

**Figure 4:**
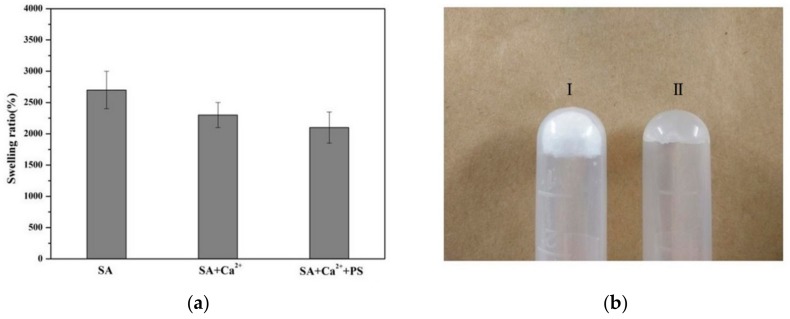
Comparisons of hydrogels (**a**) swelling ratio and (**b**) dry-wet contrast diagram.

The degree of swelling of a hydrogel was determined by its internal structure and level of crosslinking. The greater the degree of crosslinking, the denser the network formed, the smaller the amount of space storage, and the lower the degree of swelling. Conversely, the degree of swelling was higher [32]. The drug release characteristics of the hydrogel can be highly dependent on its swelling characteristics. As can be seen in Figure 4a, the degrees of swelling of SA, SA and Ca^2+^ crosslinking (SA + Ca^2+^), and TSPMAH (SA + Ca^2+^ + PS) gradually decreased, which might be due to the fact that the phospholipid microcapsules occupied the three-dimensional network structure, thereby reducing the space for the solvent storage of the gel and showing a lower degree of swelling. The degree of swelling of the hydrogel crosslinked with Ca^2+^ was slightly higher, indicating that the gel had higher internal water content, greater flexibility, and easy deformation. The dry gel of the phospholipid microcapsule composite SA (I) and the swollen hydrogel (II) were shown in Figure 4b.

### 2.2. Analysis of Drug Loading Effect of TSPMAH

**Figure 5:**
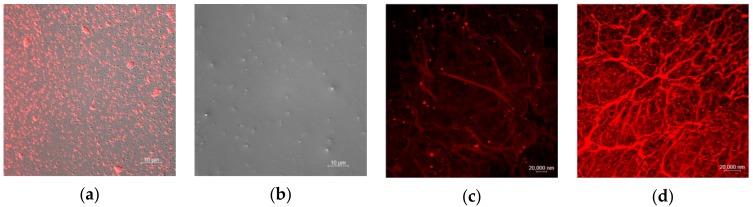
LSCM images of (**a**) protamine–siRNA complexes, (**b**) thermo-responsive phospholipid microcapsules, (**c**) drug-loaded composite hydrogel of thermo-responsive phospholipid microcapsules in concentrations of 0.1 mg mL^−1^, and (**d**) drug-loaded composite hydrogel of thermo-responsive phospholipid microcapsules in concentrations of 0.2 mg mL^−1^.

To test the thermal responsivity of the TSPMAH when carrying drugs, the protamine–siRNA complex was prepared and encapsulated in the TSL as the model drug before the TSL was grafted onto alginate hydrogel to form TSPMAH. Ultra-high-speed centrifugation was used to separate the encapsulated and free drugs. After the mixture was centrifuged at 10,000 rpm for 10 min, the supernatant was aspirated. Using the buffer as a blank control, the absorbance at 223 nm of the protamine–siRNA complex was measured with a K5800 ultra-micro spectrophotometer. According to the formula: encapsulation rate = (total dosage-free drug)/total dosage × 100%, the encapsulation rate of the thermosensitive phospholipid microcapsule was known as being 75.8%. The release of the protamine-siRNA complexes (among which the siRNA was labeled with Cy3) via temperature changes can be easily visualized under a confocal microscope. The protamine–siRNA formed by electrostatic interaction between the protamine and the phosphorus backbone of the siRNA made the protamine-siRNA hydrophobic, while the thermo-responsive phospholipid microcapsules were amphiphilic [33], the hydrophilic head was exposed in water, and the hydrophobic tail bound to protamine-siRNA, encapsulating the protamine-siRNA into TSL [34]. The drug-loaded phospholipid microcapsules were then grafted onto the alginic acids by forming a Ca^2+^ “bridge” between the carboxyl group in a PS and the carboxyl group in alginic acid. LSCM images were presented in Figure 5, the protamine-siRNA was seen as a spherical-like complex (Figure 5a), and the thermo-responsive phospholipid microcapsules were seen as uniform spherical membrane particles (Figure 5b). As can be seen in Figure 5c,d, the protamine-siRNA complexes had efficiently encapsulated into the TSPMAH dependent of the concentrations of the TSL added in the gel system. Figure 5c showed the TSPMAH prepared by adding the TSL to the final concentration of 0.1 mg mL^−1^, while Figure 5d showed the TSPMAH was prepared by adding the TSL to the final concentration of 0.2 mg mL^−1^ (Figure 5d), showing clearly with different grafting efficiencies.

### 2.3. Analysis of Drug Release of the TSPMAH

**Figure 6:**
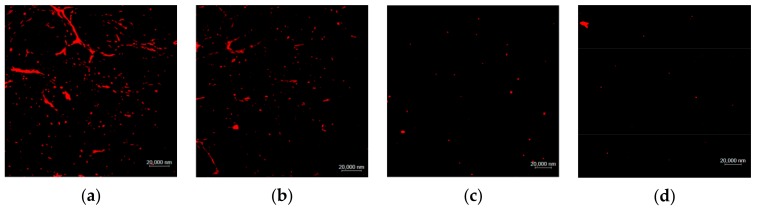
The releasing of protamine–siRNA complexes from the TSPMAHs at different temperatures. (**a**) 25 °C, (**b**) 37 °C, (**c**) 40 °C, and (**d**) 43 °C.

The water bath heating method was used to investigate the release of the drugs encapsulated in the TSPMAH in vitro at temperatures of 37 °C, 40 °C, and 43 °C, respectively. Toward this end, the TSPMAH loading with protamine-siRNA complexes was dialyzed in a water bath at 37 °C, 40 °C, and 43 °C, respectively, for 8 h. The drug-releasing was analyzed under a confocal fluorescent microscope and the results were presented in Figure 6. As it was seen in Figure 6, a small number of protamine-siRNA complexes were released from the drug-loaded TSPMAH at 37 °C (Figure 6b) when compared with those were released at 25 °C (Figure 6a), and the release turned to be much more pronounced as the temperature increased (Figure 6c,d). The UV absorptions of the liquids were then measured before and after the protamine-siRNA complexes were released from the TSPMAH by using a spectrophotometer (Table 2). As it can be seen in Table 2, the cumulative releasing rates of the protamine-siRNA complexes from the TSPMAHs were above 90% at the temperature of 43 °C (Table 2).

### 2.4. Cytotoxicity Analysis of TSPMAH

**Figure 7:**
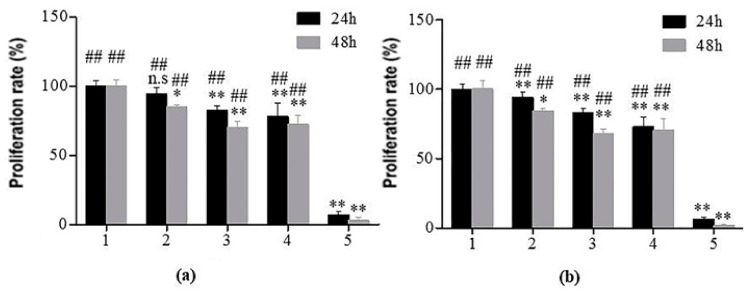
Effects of the composite hydrogel on cell proliferations. (**a**) Endothelial cells: 1, negative control, 2, SA extract, 3, TSPMAH, 4, SA, 5, positive control. * *p* < 0.05, ** *p* < 0.01 vs. negative control group; ## *p* < 0.01 vs. positive control group; n.s: nonsignificant, and (**b**) myocardial cells: 1, negative control, 2, SA extract, 3, TSPMAH, 4, SA, 5, positive control. * *p* < 0.05, ** *p* < 0.01 vs. negative control group; ## *p* < 0.01 vs. positive control group; n.s: nonsignificant.

The leach solution method was a commonly used method in cytotoxicity experiments [35]. In the method, the test substance was immersed in distilled water or culture solution under appropriate conditions to obtain an eluate of the test liquid, the eluate was added to the cells to continue the culture, and the effects of eluate on cell proliferation could be observed. In this experiment, human endothelial cells (Figure 7a) and cardiomyocytes (Figure 7b) were used for toxicological experiments of the materials to be tested. In Figure 7, 1–5 were the negative control, SA extract, TSPMAH, SA, and positive control, respectively. The cell proliferation rates of the TSPMAH were above 81% in 24 h, indicating that the material was basically non-toxic (*p* > 0.05) and could be used as a biomedical drug delivery material. The test of a single component of thermo-responsible phospholipid microcapsules and SA did not show significant cytotoxicity, and all cell viabilities were shown to be above 69%, indicating that phospholipid microcapsules and SA had good biocompatibility and low toxicity (*p* < 0.05). However, we did notice a slight decrease in the proliferation rates of the endothelial cells and myocardial cells in the 24 h incubation and in the 48 h incubation, respectively. In addition, the concentration of the single component was slightly higher, which might somehow interfere with the cell proliferation (*p* < 0.01).

## 3. Materials and Methods 

### 3.1. Chemicals and Materials

siRNA: 5′-Cy3-UGAUGCUGGGCUGAGUAAGUUAGGAUU-dTdT-3′ was synthesized by Sangon Biotech Co., Ltd. (Shanghai, China). DPPC (1,2-dipalmitoyl-sn-glycero-3-phosphocholine), sodium alginate (SA), CaCl_2_, *N*-2-hydroxyethylpiperazine-*N*-ethane-sulphonicacid (HEPES), dimethyl sulfoxide (DMSO), and phenol were purchased from Aladdin Co., Ltd. (Shanghai, China). Phosphatidylserine (PS) was purchased from Avanti^®^ Polar Lipids, Inc. (Alabaster, AL, USA). Polyethylene glycol stearyl ether (Brij78), protamine, and Dulbecco’s modified eagle medium (DMEM) cell culture solution were purchased from Sigma-Aldrich Co. (St. Louis, MO, USA). Human endothelial cell line Ealy-926 and rat cardiomyocytes cell line H9c2 were purchased from Laboratory Animal Center, Sun Yat-sen University, Guangzhou, China. All the experimental reagents were of analytical grade, and the water used in the experiments was sterilized and steamed water three times.

### 3.2. Preparation of TSPMAH

Thermosensitive phospholipid microcapsules were prepared by using a membrane dispersion method as follows [36,37]. DPPC, PS, and Brij78 were dissolved in chloroform at a molar ratio of 96:0:4, 94:2:4, 92:4:4, 90:6:4, and 88:8:4, respectively. Each solution was slowly dried with nitrogen until a thin-film was formed. The film was placed in a vacuum desiccator to remove the residual chloroform for 2 h, and hydrated in 3 mL of HEPES buffer at room temperature for 1 h. The sample was sonicated for 8 min at 60 w, 90 w, 120 w, 150 w, or 180 w using a cell pulverizer on an ice bath. To increase the stability of the thermosensitive phospholipid microcapsules, CaCl_2_ (pH = 7.4) was added to a final concentration of 0 mM, 10 mM, 20 mM, 30 mM, and 40 mM, respectively. 

To prepare TSPMAH, 1% SA was added dropwise to the thermo-responsible phospholipid microcapsule system, and the mixture was stirred uniform dispersion of the thermo-responsible phospholipid microcapsules in the SA gel. The concentration of SA in the mixture was maintained at 0.33%. Then, the mixture was centrifuged at 4500 r/min for 10 min, and the precipitate was composite hydrogel (TSPMAH). 

### 3.3. Measurements of the Zeta Potentials of Phospholipid Microcapsules

The zeta potentials were measured using a Zeta Potential Analyzer (Beckman Coulter, Pasadena, CA, USA). CaCl_2_ (300 mM, pH = 7.4) was used to improve the stability of the thermosensitive phospholipid microcapsules in HEBES buffer. The concentration of CaCl_2_ was added to the final concentrations of 0 mM, 10 mM, 20 mM, 30 mM, and 40 mM, respectively, and the optimal stability of the thermosensitive phospholipid microcapsules system was screened by differential scanning calorimetry. Each sample was measured three times in parallel.

### 3.4. FTIR Characterizations of the TSPMAH 

The interactions between the liposome and the SA were analyzed by Fourier transform infrared spectroscopy using a Nicolet FTIR spectrometer (Thermo Fisher Scientific, Waltham, MA, USA). The samples were ground carefully with KBr and then pressed into disks for observation, and the scanning range was 4000 cm^−1^ to 500 cm^−1^.

### 3.5. SEM and TEM Observations of TSPMAH

For TEM, the synthesized materials were dropped onto a 300-mesh carbon grid, stained with 2% phosphotungstic acid for 3 min, and then freeze-dried, high-resolution TEM analysis was carried out on a Tecnai G2 F20 S-TWIN transmission electron microscope (FEI, Hillsboro, OR, USA) at an accelerated voltage of 120 kV. 

The SEM observation on the synthesized materials was carried out as follows: 10 μL of the prepared materials were dropped onto a treated silicon wafer and vacuum-dried for half an hour. The conductive adhesive was applied for the fixation of the dry materials. After the surface of the material was sprayed with gold, the SEM images were obtained by using a JSM-7500F Cold field emission scanning electron microscope (JEOL, Chiyoda, Tokyo, Japan).

### 3.6. DSC Analysis of the Thermo-Responsibilities of the Phospholipid Microcapsules 

The Tm values were determined by a DSC Q2000 differential scanning calorimeter (TA Instruments, New Castle, DE, USA). Of the prepared materials, 10 μL was dropped onto the aluminum pan. After weighing, it was sealed and placed in the sample tank. At the same time, an empty aluminum pan was set as a reference and placed in a reference tank. The temperature range was from 25 °C to 55 °C, and the initial equilibration time was 2 min. To ensure that the sample maintained a relatively balanced state during the gradual heating process, the heating rate was selected to be 1 °C/min.

### 3.7. Sizing of the Thermo-Responsible Phospholipid Microcapsules 

The sizes of the thermo-responsible phospholipid microcapsules were analyzed by a Delsa Nano C nanoparticle Size and Zeta Potential analyzer (Beckman Coulter Life Sciences, Indianapolis, IN, USA). The hydrated thermosensitive phospholipid microcapsules were firstly mixed using a homogenizer. The ultrasonic probe of the cell crusher was placed in the center of the centrifuge tube to prevent the probe from sticking to the wall. The thermosensitive phospholipid microcapsules were ultrasound in an ice bath for 8 min and then changed the ultrasound power to 60 w, 90 w, 120 w, 150 w, and 180 w, respectively. The changes in the size of the thermosensitive phospholipid microcapsules were observed and recorded.

### 3.8. The Rheological Properties of the TSPMAH

The rheological properties of the TSPMAH, including the frequency scanning and temperature phase transition, were studied by using an AR2000ex rotary rheometer. To do this, a cone-and-plate fixture having a cone angle of 1° and a diameter of 40 mm was used. In order to erase any previous shear histories and ensure the equilibrium structure, a steady preshear was applied at a shear rate of 1 s^−1^ for 60 s, followed by a 120 s rest period before any dynamic experiments. The measurements were conducted at 35 °C, 40 °C, and 45 °C, respectively.

### 3.9. Drug Loading and Drug Release of TSPMAH

Spherical shaped protamine-siRNA complexes were prepared using 5′-Cy3-labeled siRNA by following a protocol previously published by our laboratory [38]. The protamine-siRNA complexes were then encapsulated into the TSPMAH. In vitro release of drugs loaded by TSPMAH at different temperatures was treated by dialysis of the protamine–siRNA complex loading TSPMAH in HEPES buffer at 37 °C, 40 °C, and 43 °C for 8 h; the blank controls were dialyzed at 25 °C for 8 h. The drug loading efficiencies of different concentrations of thermo-responsible phospholipid microcapsules and the drug release of the protamine–siRNA complex loading TSPMAH were investigated by an LSM880 laser scanning confocal microscope (Zeiss, Oberkochen, Germany). 

### 3.10. Determination of the Swelling Ratio of the Composite Hydrogel

The swelling ratio (SR) of the composite hydrogel was determined by a quality method. First, equal volumes of SA, Ca^2+^ crosslinked SA and TSPMAH were dried in a vacuum freeze dryer for 8 h in centrifuge tubes, and 4 mL of HEPES buffer was added to each centrifuge tube for swelling for 48 h. The SR of the hydrogel was calculated by the following formula [39]:

(1) $$SR=\frac{W_{2}-W_{0}}{W_{1}-W_{0}}\times 100\%$$

In Formula (1), the W_0_ was the mass of the centrifuge tube, W_1_ was the total mass of the dried hydrogel and the centrifuge tube, and W_2_ was the total weight of the swollen hydrogel and the centrifuge tube.

### 3.11. Cytotoxicity Study of TSPMAH

In vitro cell culture and MTT assay were conducted to analyze the cytotoxicity of TSPMAH. Human endothelial cell line Ealy-926 and rat cardiomyocytes cell line H9c2 were used by this study. To obtain the extracts of TSPMAH, TSPMAH was added into liquid DMEM medium and incubated at 37 °C for 24 h in the presence of 5% CO_2_. Then, supernatants were collected, centrifuged, and filtered to remove any debris. Meanwhile, the hydrogel group, SA group, phospholipid microcapsules group, blank control group, and positive control group were set up on 96-well culture plates, and the endothelial cells and cardiomyocytes were seeded into each well by a seeding amount of ~5000 cells/well, respectively. Then, 100 μL of the supernatants of the TSPMAH extracts was added into wells in the hydrogel group, and 150 μL of sodium alginate solution (0.2%), microcapsule stock solution and DMEM medium was added into the wells in the SA group, the phospholipid microcapsules group, and the blank control group, respectively. After that, 5 g/L phenol solution was added into the wells in the positive control group. Incubations were conducted for 24 h and 48 h, respectively. The morphology of the cells in each group was observed under a microscope. After the microscopy observations, 20 μL of 5 g/L MTT solution was added to each well and further cultured for 4 h, and then the supernatants in the wells in each group were removed, 150 μL DMSO was added into each well, and the UV absorbance of each solution was measured at 570 nm on an ELx800 enzyme-labeled instrument (Olympus, Shinjuku City, Tokyo, Japan).

The relative cell viability (R) was calculated using a method reported by Chang as listed in Formula (2) [40].

(2) $$R(\%)=\frac{A(test)}{A(control)}\times 100\%$$

In Formula (2), the *A* (test) was the absorbance of the cells to be tested, and the *A* (control) was the absorbance of the control cells.

### 3.12. Statistical Analysis

Data collected in the cytotoxicity study of the TSPMAH were presented as the means ± SEM. Statistical significance was determined by using the one-way ANOVA for multiple comparisons, and the Student’s *t*-test of the SPSS22.0 software was used for comparing the mean differences between every two groups. *p* < 0.05 was considered significant. 

## 4. Conclusions

In summary, a type of temperature-responsive phospholipid microcapsules as-prepared by optimizing the molar ratio of DPPC, PS, and Brij78. Further, a novel type of temperature-responsive phospholipid microcapsule-SA composite hydrogel was prepared by encapsulation of the phospholipid microcapsules in the SA hydrogel via the formation of PS-Ca^2+^-carboxyl-SA bridge. The composite hydrogel exhibited a regular spherical shape and was highly cytocompatible with human endothelial cells and myocardial cells. The composite hydrogel showed a controlled release of the loaded protamine-siRNA complexes by following temperature changes. 

## Figures and Tables

**Table 1:** Particle sizes of thermosensitive phospholipid microcapsules at different concentrations of Ca^2+^.

[Ca^2+^] (mM)	0	10	20	30	40
D (90%) (d. nm)	224.6 ± 3.2	221.5 ± 1.9	226.2 ± 4.3	223.7 ± 2.5	220.3 ± 2.7
Average Size (d. nm)	124.3 ± 1.1	125.5 ± 2.4	126.2 ± 3.7	133.8 ± 2.8	127.6 ± 3.0

**Table 2:** The cumulative releasing rates of protamine-siRNA complexes from the TSPMAHs versus temperatures.

Temperature (°C)	25	37	40	43
Protamine–siRNA complexes releasing rates (%)	26.32	45.08	82.56	92.15
